# Asymmetry of generalized discharges in idiopathic generalized epilepsy in adults

**DOI:** 10.1111/epi.18509

**Published:** 2025-06-21

**Authors:** Joao Pizarro, Matthew C. Walker, Laurent Sheybani

**Affiliations:** ^1^ Chalfont Centre for Epilepsy, University College London Hospitals NHS Foundation Trust London UK; ^2^ Department of Clinical and Experimental Epilepsy UCL Queen Square Institute of Neurology, University College London London UK; ^3^ National Hospital for Neurology and Neurosurgery, University College London Hospitals NHS Foundatin Trust London UK; ^4^ NIHR University College London Hospitals Biomedical Research Centre London UK

**Keywords:** amplitude, discharges, EEG, epilepsy, generalized, symmetry

## Abstract

Generalized epileptiform discharges (GEDs) in idiopathic generalized epilepsy (IGE) are classically considered symmetrical in amplitude, although this has not been formally tested. This is a major knowledge gap, since asymmetry is conventionally considered an atypical feature, with clinical implications. Furthermore, if such asymmetry exists, it would challenge the concept that IGE engages homogenously the entire brain and rather supports the hypothesis that IGE is shaped by delimited networks. Here, we asked whether GEDs in IGE are asymmetrical and whether this asymmetry exhibits individual specificity. Across 62 patients with IGE recorded with scalp electroencephalography (EEG), we found that GEDs exhibit hemispheric asymmetry (mean, 95% confidence interval [CI]: 22%, 17%–28%) in comparison to control conditions and preceding baseline. Importantly, this asymmetry is systematic across several repetitions of GEDs. Furthermore, the asymmetry varies more across than within patients, indicating that it operates as an individual trait. In contrast, there was no left–right hemispheric preference, and the asymmetry was not different when comparing wake‐ and sleep‐recorded GEDs. Beyond the clinical relevance of providing a formal range for GEDs asymmetry in IGE (upper range of 95% CI ~30% asymmetry), this work supports the concept of IGE as being shaped by distinct and lateralized cortical brain networks.

**Key points**

- Generalised epileptiform discharges often exhibit asymmetrical amplitude in adults with idiopathic generalised epilepsy and should not be considered an atypical feature.
- The upper end of the 95% confidence interval is ~30% asymmetry of amplitude.
- This asymmetry is not different during wakefulness and sleep.
- This asymmetry is highly patient‐specific and supports the hypothesis that generalized discharges are shaped by delimited areas of cortex.

## 1 INTRODUCTION

In idiopathic generalized epilepsy (IGE), epileptiform discharges are classically regarded to engage the whole brain simultaneously. However, recent evidence indicates that focal regions may initiate epileptic activities that rapidly generalize,^1^, ^2^ and topological reorganization of brain networks has been observed in people with IGE.^3^ The involvement of delimited brain regions as drivers of epileptic activities in IGE may manifest as distinct focal characteristics of generalized epileptiform discharges (GEDs), but currently evidence is lacking.

Asymmetry of GEDs has been reported,^4^ but it is unclear whether the asymmetry is noted on a few discharges or systematically across multiple ones.^5^, ^6^, ^7^, ^8^, ^9^ Moreover, it is uncertain what constitutes an acceptable degree of asymmetry and whether such asymmetry reflects that of background activity,^5^, ^6^, ^10^ which would suggest that it is not specific to GEDs.^11^, ^12^ Furthermore, the threshold to qualify a GED as asymmetrical is often defined arbitrary,^5^, ^6^, ^7^, ^8^, ^9^, ^10^ making it challenging to test its reproducibility across GEDs and across studies. Altogether, a formal quantification of GED amplitude asymmetry is lacking.

This is a major gap in clinical and basic scientific knowledge. Indeed, the level of asymmetry is used in clinic—among other parameters—to confirm or refute the diagnosis of IGE.^4^, ^13^ From a mechanistic perspective, identifying asymmetry would fit with experimental evidence of asymmetric cortical regions exhibiting changes in activity before ictal discharges in models and people with IGE.^1^, ^2^ A systematic asymmetry of activity during GEDs, that is, across several GEDs but not during baseline, would indicate that GEDs are not homogenous, but constrained by individual networks.

Here, we tested in a cohort of 62 patients with IGE the reproducibility of GED asymmetry across several GEDs. We computed the variability of this asymmetry within patients and across patients to assess the individuality of this trait. Finally, we compared wake and sleep data to identify the potential impact of vigilance state.

## 2 METHODS

### 2.1 Data and participants

We retrospectively identified 70 sequential adult patients with a putative diagnosis of IGE who had scalp electroencephalography (EEG) demonstrating GEDs during routine, ambulatory, or video‐EEG telemetry recordings as part of their clinical assessment (Table 1). Eight patients were excluded due to a dual diagnosis of focal and generalized epilepsy, non‐IGE epilepsy syndrome, or the presence of a focal lesion on magnetic resonance imaging (MRI). Probable cases were those for whom the treating physician expressed some uncertainty of the diagnosis. This uncertainty was often motivated by an asymmetry of GED amplitude, and excluding these patients would have thus been circular given the focus of the current study. Analyses were performed on definite and probable cases altogether, and subsequently on definite cases only. EEG recordings were acquired using SystemPlus software from Micromed, using a modified 10–20 montage with the addition of sphenoidals and mastoid electrodes. Sampling rate was 256 Hz (512 Hz in eight patients) with a bandpass filter at 0.53–70 Hz and 50 Hz notch filter. FCz and CPz were used as reference and ground, respectively. The data set comprised previously collected routine clinical data, including de‐identified EEG data, and was approved by the National Hospital for Neurology and Neurosurgery Research Ethics Committee as a service evaluation; individual consent was, therefore, not required.

**TABLE 1:** Patients demographics.

S	Diagnosis	EEG type	#	Sex	Age	Hand	Duration	Treatment	Status
1	Absence	Telemetry	94	F	18	R	7 months	LTG	P
2	Absence	Ambulatory	39	M	52	R	42 years	LEV, LCM, ZNS, CLB, DZP (PRN), GBP	D
3	Jeavon	Telemetry	1010	F	26	R	17 years	BRV, CNB, ETS, LCM, CLB (PRN), LZP (PRN), MDZ (PRN)	D
4	Absence	Ambulatory	223	F	31	R	20 years	CLB, LTG, TPM, LCM	D
5	Jeavon	Telemetry	67	F	35	R	16 years	ZNS	D
6	Absence	Telemetry	45	F	35	R	30 years	ESL, PER	P
7	Absence	Telemetry	50	F	40	R	32 years	LTG, ZNS, CLB, PGB	D
8	Absence	Telemetry	45	M	26	R	12 years	LEV, CLB, MDZ (PRN)	D
9	Absence	Telemetry	32	F	19	R	7 years	VPA, ZNS	D
10	Absence	Telemetry	52	M	28	R	17 years	MDZ, LCM, LEV	D
11	Jeavon	Ambulatory	56	F	24	L	24 years	BRV, CNB, CLN, CLB (PRN), CLN (PRN)	D
12	Jeavon	Telemetry	94	F	29	R	18 years	ZNS, LCM, CLN	D
13	Absence	Ambulatory	43	F	52	R	45 years	ETS, LTG, CLB (PRN)	D
14	Absence	Telemetry	480	F	21	R	8 years	CLN, LTG, ZNS	D
15	Jeavons	Telemetry	91	F	18	R	4 years	TPM, PER, CLB	D
16	Absence	Telemetry	54	M	63	R	50 years	LTG, LEV, PER	D
17	Absence	Telemetry	11	F	18	R	16 years	ESL, ZNS	D
18	Absence	Telemetry	46	M	61	R	56 years	VPA, CLB (PRN)	D
19	Absence	Telemetry	20	F	53	R	45 years	LTG, CLN, OXC, CLB, MDZ (PRN)	P
20	Absence	Telemetry	43	M	22	L	5 years	LCM, VPA, ZNS	D
21	Absence	Telemetry	56	F	39	R	n/a	LTG	D
22	Absence	Telemetry	8	F	17	R	13 years	ZNS	D
23	JME	Ambulatory	19	M	43	R	28 years	LTG, VPA, ZNS	D
24	Absence	Telemetry	233	F	29	R	25 years	LEV	D
25	JME	Telemetry	158	F	62	R	49 years	CBZ, ZNS	D
26	JME	Telemetry	153	F	20	R	9 years	LEV, OXC	D
27	Absence	Telemetry	40	F	18	R	6 years	LTG, PER, MDZ (PRN)	P
28	JME	Sleep	13	F	26	R	Unclear	PB, LTG, CLB	D
29	JME	Telemetry	75	F	38	L	25 years	ZNS, LTG, CLB	P
30	Absence	Routine	21	F	34	R	14 years	ESL, LEV, LCM	P
31	JME	Routine	3	M	18	R	n/a	Nil	D
32	Absence	Routine	5	F	51	R	n/a	LTG	D
33	GTCA	Ambulatory	9	M	21	R	n/a	VPA, LTG, CLB, LEV	P
34	Absence	Ambulatory	95	F	23	R	2 years	LTG	D
35	JME	Telemetry	68	M	24	R	6 years	VPA	D
36	JME	Telemetry	72	M	21	L	13 years	BRV, LCM, ETS	D
37	GTCA	Telemetry	23	M	40	R	25 years	LTG, VPA, MDZ (PRN)	D
38	JME	Telemetry	43	F	37	R	26 years	PER, LEV	P
39	Absence	Telemetry	48	M	21	R	8 years	LEV, LTG	D
40	JME	Telemetry	12	F	27	R	8 years	LTG, OXC, ZNS, MDZ (PRN)	D
41	JME	Telemetry	59	F	27	R	11 years	LEV	P
42	JME	Telemetry	61	M	46	R	30 years	LEV, PB, PHT	P
43	JME	Routine	8	F	17	n/a	n/a	n/a	P
44	Absence	Routine	7	F	18	R	11 years	LTG	D
45	Absence	Routine	6	F	17	R	n/a	Nil	P
46	Absence	Telemetry	86	F	40	R	30 years	LTG, BRV, PER, CLB (PRN)	D
47	Absence	Telemetry	28	F	22	R	8 years	LCM, PER	D
48	JME	Telemetry	25	F	25	R	13 years	TPM, LTG	D
49	JME	Ambulatory	61	F	26	R	17 years	LTG, PER, LEV, CLB	D
50	JME	Telemetry	34	F	41	R	33 years	VPA, CLN, ZNS	D
51	Jeavon	Prolonged	102	F	17	R	11 years	ZNS	D
52	Absence	Ambulatory	165	F	16	R	10 years	LEV	D
53	GTCA	Telemetry	33	M	20	R	8 years	LEV, CLB	P
54	JME	Telemetry	13	F	17	R	1 year	MDZ (PRN)	P
55	GTCA	Routine	27	M	48	a	31 years	ZNS, LTG	P
56	GTCA	Routine	14	F	46	L	Unclear	CBZ	P
57	JME	Ambulatory	61	F	38	R	Unclear	LEV	P
58	JME	Routine	12	F	22	n/a	n/a	Nil	P
59	JME	Routine	4	F	24	L	11 years	VPA, CLB (PRN)	D
60	Absence	Telemetry	19	M	54	R	47 years	VPA	P
61	Absence	Telemetry	27	F	23	R	4 years	ZNS, DZP, LEV, MDZ (PRN)	D
62	Absence	Telemetry	97	F	35	R	18 years	LEV, LCM	D

*Note*: From the 11 routine EEGs, 5 had sleep episodes from which ≥1 GED.

Abbreviations: #, number of GED (wake+sleep); a, ambidextrous; CLB, clobazam; CLN, clonazepam; CNB, cenobamate; D, definite diagnosis; Duration: duration of epilepsy; ESL, eslicarbazepine; ETS, ethosuximide; GBP, gabapentine; Hand, handedness; LCM, lacosamide; LEV, levetiracetame; LTG, lamotrigine; P, probable diagnosis; PER, perampanel; PGB, pregabaline; PHT, phenytoin; PRN, on demand; S, subject; Sex, biological sex. BRV, brivaracetam; TPM, topiramate; VPA, valproate; ZNS, zonisamide.

### 2.2 Identification of GEDs

Epileptiform discharges were identified using Cartool for display^14^ by neurophysiologist J.P. and epileptologist L.S. following published guidelines.^13^ Epileptiform discharges were defined as generalized spike–wave or poly‐spike–wave discharges (henceforth generalized epileptiform discharges [or GEDs]) within 2.5–5.5 Hz. When possible, at least 10 GEDs during sleep and wake were saved for analysis.

### 2.3 Hemispheric asymmetry computation

Data were re‐processed into an average reference montage for analyses. Amplitude was measured on F3, F4, C3, and C4, since those electrodes display the most prominent GEDs.^15^ Amplitudes were obtained through Hilbert transform of the filtered signal between 2 and 4 Hz (order 2 Butterworth filter). Median amplitude was computed by hemisphere and then averaged across individual GED duration. One asymmetry ratio for each GED was calculated as the ratio between hemispheres. Then, within participants, the median asymmetry across GEDs was saved as the individual asymmetry. Because we were interested in any asymmetry in this analysis (left > right or right > left), this value was flipped to be >1 on an individual basis. For control, we computed the asymmetry ratio after randomly shuffling the right and left hemispheres across 10 000 permutations. It is notable that at each permutation, we flipped the surrogate median asymmetry to >1 to follow the same computation as for the unshuffled data (Figure S1).

For the pre‐GED epoch, we computed the median asymmetry following the same protocol but focusing on the period from −7 to −2 s before the onset of the GEDs. This was based on the fact that we wanted to include sufficient pre‐GED data to assess reliably the baseline, while also using a range close to that of GED duration (across patients and vigilance states: 2.5 s, range: 0.5 to 14 s). The median asymmetry before GEDs was flipped (i.e., 1/median asymmetry) only if the median asymmetry during GEDs was also flipped to be >1. This ensured that any bias for one hemisphere during GEDs would be compared to the same bias before the GEDs.

### 2.4 Right–left asymmetry computation

We followed the same strategy as above, except that the asymmetry ratio was never flipped. In this analysis, values >1 indicate an asymmetry in favor of the right hemisphere.

### 2.5 Computation of the asymmetry as an individual trait

To test whether the variability, lateralization, and extent (i.e., amplitude) of asymmetry was more variable within than across patients, we first computed the coefficient of variation of the asymmetry across all GEDs within patients, hereafter: ”within coefficient of variation (CV).” Then, across 10 000 permutations, we shuffled all individual asymmetry indices and randomly selected for each patient as many GEDs as they had initially. We then computed a surrogate CV per patient and eventually the averaged surrogate CV across permutations, henceforth ‘surrogate CV’ or ‘CV across’.

### 2.6 Statistics

Analyses were performed in Matlab (version 2024b) and statistics in GraphPad Prism (version 10.4.1) and Jamovi (version 2.6.22). To test any hemispheric or right–left asymmetry, we ran mixed‐effects analyses with factor 1 vigilance (two levels: wake vs sleep) and factor 2 condition (three levels: during GED, during GED with shuffled left and right hemispheres, before GED). To compare the CV within vs across patients, we used a mixed‐effects analysis using factor 1 vigilance and factor 2 condition (two levels: within, across). For post hoc analyses, we averaged wake and sleep data, based on a lack of vigilance × condition interaction, and performed a one‐way analysis of variance (ANOVA) across the levels of factor “condition.” Several patients had >2 antiseizure medications (ASMs). Hence, we performed a subanalysis to differentiate patients with ≤2 and those with >2 ASMs using a linear mixed model:

$$\begin{array}{c} \text{asymmetry}\sim \text{condition}+\mathrm{ASM}+\text{vigilance}+\text{condition}\\ \times \text{vigilance}+\text{condition}\times \mathrm{ASM}+\text{vigilance}\times \mathrm{ASM}\\ +\text{vigilance}\times \mathrm{ASM}\times \text{condition}+(1\;|\;\text{subject}). \end{array}$$

where “condition” had three levels (during GED, during GED with shuffled left and right hemispheres, before GED), “ASM” had two levels (≤2 ASMs vs >2 ASMs), and “vigilance” had two levels (wake vs sleep).

## 3 RESULTS

### 3.1 Hemispheric asymmetry

**FIGURE 1:**
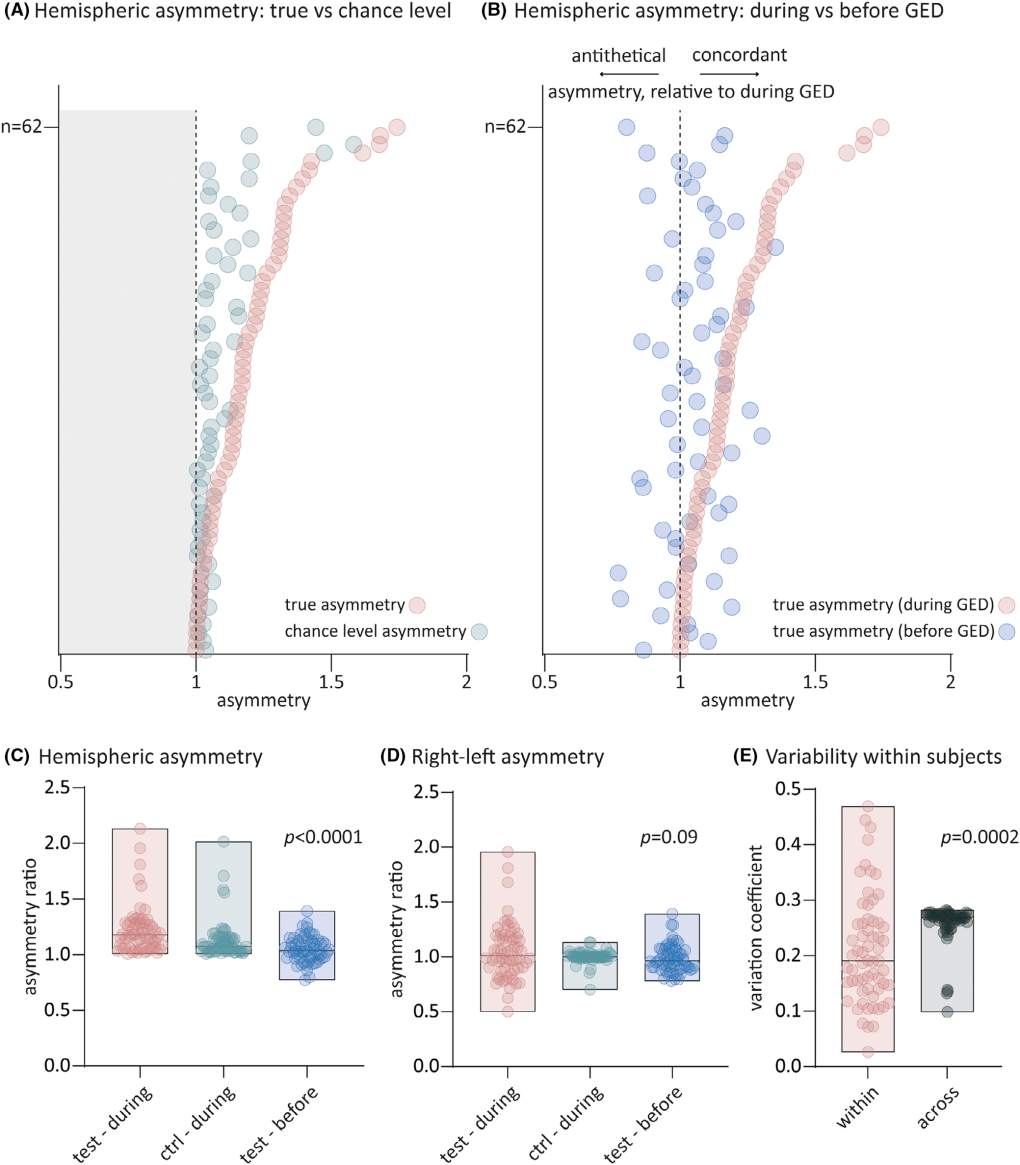
Hemispheric asymmetry in IGE is an individual trait. (A, B) True (pink dots), chance level (GED with randomized right and left hemisphere, green dots), and asymmetry before GEDs (blue dots). Patients (1–62) are organized along increasing values of true asymmetry. (A) The asymmetry indices are flipped to values >1 in both conditions but the true asymmetry indices are systematically higher than that of controls. The gray area on the left reflects the fact that this procedure cannot lead to asymmetries <1. (B) Asymmetry indices before GED are flipped only when they are also flipped during GED, in order to test whether any asymmetry is similar during and before GED. As seen, the asymmetry bias before GEDs is different than that during GEDs, both in terms of amplitude, that is, line‐wise (pink and blue dots are not equally away from the 1 dashed line); and in terms of lateralization, that is, 21 blue dots (34%) are on the left side of the one dashed line, reflecting antithetical asymmetry before GEDs, in comparison to during GEDs, for these 21 patients. (C) The hemispheric asymmetry is significantly higher than the chance‐level (green) and before GED (blue). (D) There is no significant right–left asymmetry. (E) We found a significantly lower variability of GED asymmetry within than across patients. The *p*‐value indicates the main effect of condition. For display, data are combined across sleep and wake given the lack of effect of vigilance. Boxes: min to max, line at median. Dots: individual subjects.

We first assessed the presence and degree of any asymmetry, that is, in the right or left hemisphere. The consistency of asymmetry was assessed by comparing the median asymmetry of GEDs against that of GEDs with randomized left and right hemispheres (see Figure S1 and Methods). We also compared GED asymmetry against that of the preceding baseline, to test if any asymmetry was specific to GEDs. We hence had three conditions (during GEDs, during GEDs with randomized hemispheres, before GEDs) and two vigilance states (wakefulness and sleep). We found a significant main effect of condition (*F*(1.2,72) = 20.1, *p* < .0001, Figure 1A–C) on hemispheric asymmetry. Post hoc analysis indicated that GED asymmetry level was significantly higher than chance (mean difference, 95% confidence interval [CI]: 9%, 7%–12%; *p* < .0001), and than asymmetry before GEDs (17%, 10%–25%; *p* < .0001, Figure 1B,C). We obtained similar results using definite cases only. Indeed, we found a significant main effect of condition (*F*(1.2,51.7) = 16.0, *p* < .0001, Figure S2A). Post hoc analysis indicated that GED asymmetry was significantly higher than chance (mean difference, 95% CI: 10%, 6%–14%; *p* < .0001), and than asymmetry before GEDs (17%, 8%–26%; *p* = .0002, Figure S2A). We also removed patients with extreme numbers of GED (< or > to the interquartile range [IQR]) and, again, observed a significant effect of condition (*F*(1.18,26.03) = 7.92, *p* = .007, post hoc: during GEDs vs during GEDs with randomized hemispheres: *p* < .0001; during GEDs vs before GEDs: *p* = .02, Figure S2B). Finally, using a linear mixed model to differentiate the effect in patients with ≤2 and >2 ASMs, we again found only an effect of condition, without interaction with ASMs. The asymmetry during GEDs (mean ± standard error: 1.22 ± 0.02) was significantly larger than chance level (1.12 ± 0.02, *p* = .001, Bonferroni corrected), and than that before GEDs (1.05 ± 0.02, *p* < .001, Bonferroni corrected) (fixed‐effect Omnibus test, main effect of condition: *F*(3,124.4) = 542, *p* < .001; ASM: *F*(1,39.4) = 1.7, *p* = .2; vigilance: *F*(1,198) = 0.25, *p* = .62; condition × ASM: *F*(2,187) = 1.41, *p* = .25; condition × vigilance: *F*(2,187) = 0.98, *p* = .38; ASM × vigilance: *F*(1,198) = 0.09, *p* = .77; condition × vigilance × ASM: *F*(2,187) = 0.34, *p* = .71, Figure S2C).

### 3.2 Right–left asymmetry

We found no main effect of condition for right–left asymmetry (*F*(1.4,86.1) = 2.8, *p* = .09, Figure 1D), or for condition × vigilance interaction (*F*(1.5,61.8) = 1.7, *p* = .20), indicating no general bias to one hemisphere. The same results were obtained analyzing definite cases only (Supplementary Information and Figure S3).

### 3.3 Individuality of the asymmetry

We then tested how consistent the degree of asymmetry is by comparing the coefficient of variation of asymmetry within against between patients. Using a two‐way ANOVA (factor 1 vigilance: variability during wake vs sleep; factor 2 condition: variability within vs across patients), we found that GED asymmetry is less variable within than between patients, as illustrated by a main effect of condition (*F*(1,61) = 15.4, *p* = .0002, Figure 1E). We also found a significantly lower variability of GED asymmetry within than across patients with definite IGE (main effect of condition: *F*(1,42) = 17.4, *p* = .0001). Of interest, when adding the number of ASMs as a 2 levels factor (≤2 vs >2 ASMs), we observed a significant condition × ASMs interaction (*F*(1,112.6) = 10.8, *p* < .001), indicating that people with >2 ASMs have more homogenous EEG lateralization (Figure S4).

## 4 DISCUSSION

Our work establishes that GEDs in IGE exhibit a consistent asymmetry of amplitude (Figure 1). Ninety‐five percent of our population exhibited an asymmetry within 17%–28%. In an even more conservative classification of IGE, that is, across the definite cases of IGE, the interval ranged similarly from 16% to 29%. Because these patients were diagnosed with a classical form of IGE, this indicates that asymmetry up to an increase of ~30% in IGE would not be sufficient to qualify as an atypical feature. GED asymmetry did not present with a clear right or left bias (Figure 1D).

The sleep–wake cycle has been shown to affect different features of IGE, such as the incidence of GEDs, which are more frequent during non‐rapid eye movement sleep stage 3 (NREM 3),^16^ as well as upon sleep onset, whereas less likely at sleep offset.^17^ In addition, the morphology has been shown to change during sleep, for example, irregular and poly‐spike wave discharges during NREM 3.^15^ However, despite considerable evidence of interactions between epilepsy and sleep,^18^, ^19^, ^20^, ^21^ we found no impact of wakefulness or sleep on GED asymmetry. Although our cohort is relatively large, further studies are needed to confirm the reproducibility of our findings. Our patients were identified in a tertiary epilepsy center, and care should thus be taken when extrapolating our findings to the broader population of patients with IGE.

The difference between within‐ and across‐patient consistency in patients with ≤2 vs >2 ASMs is intriguing. One could hypothesize that patients with multiple treatments constitute an independent group, perhaps due to greater drug resistance, which affects 20% of people with IGE^13^ and up to 30% of people with juvenile myoclonic epilepsy.^22^ Alternatively, this difference could result from an impact of medication on GED morphology. However, evidence of an impact of ASMs on GED morphology is limited, with one study reporting reduced propagation of discharges with increasing levels of valproate^23^ and another indicating increased amplitude ratio of the wave to spike during wakefulness under valproate, in comparison to before ASM.^24^ In rats, it was shown that multiple ASMs can lead to neurodegeneration,^25^ lending further credence to a possible drug effect.

Our results are consistent with the hypothesis that generalized discharges are shaped by delimited areas of cortex. Past studies had shown evidence that discharges might be driven by focal areas both in experimental epilepsy^1^, ^26^, ^27^ and humans with IGE.^2^ In our data, identifying the onset of GEDs would be a way to study the same mechanisms.^28^ However, much care should be given to how “onset” is defined, the risk being of a circular analysis, where asymmetric onset reflects only an ill‐defined onset. This should thus constitute an independent study with a robust and validated approach to define “onset.” The lower variability of asymmetry within than across patients (Figure 1E), together with the fact that we did not identify a bias for the left or right hemisphere (Figure 1D), suggests that the asymmetry we observed is not driven by “hardware” constraints shared across subjects as are, for example, language (left hemisphere), face recognition (right hemisphere),^29^ or the posterior dominant rhythm (higher amplitude on the right, non‐dominant, hemisphere).^30^ However, individual specificities in brain network might contribute. In line with this, it is known that subsets of patients with IGE can display gray^31^ or white^32^ matter abnormalities. These individual differences could shape individual asymmetry of GEDs.

Asymmetries in biological systems, including the human brain,^18^, ^33^ are ubiquitous and could thus lead to an overall asymmetry in brain activity, including that during GEDs. To control for this, we compared the asymmetry during GEDs against that during the preceding baseline (Figure 1B,C). The finding that the asymmetry during GEDs is significantly more marked than asymmetry in the activity preceding GEDs suggests that this asymmetry is specific to GEDs.

Altogether, our work provides a range of asymmetry level that is seen in an IGE population, supports the concept of delimited epileptic network in IGE, and opens further questions regarding the underlying mechanisms of this asymmetry.

## AUTHOR CONTRIBUTIONS

Study design, screening for patients, data analyses, and writing—review and editing: J.P., M.C.W., and L.S. Writing—original draft: L.S.

## FUNDING INFORMATION

This work was funded by a postdoc.mobility grant from the Swiss National Science Foundation (grant P500PM_206720) and a Guarantors of Brain fellowship to L.S. This work was supportedby National Institute for Health Research (NIHR) University College London Hospital's Biomedical Research Centre.

## CONFLICT OF INTEREST STATEMENT

M.W. has acted as a consultant for Seer and EpilepsyGtx. He is a founder shareholder in EpilepsyGtx. He has received honoraria from Eisai, Angelini, and UCB pharma. The remaining authors have no conflict of interest.

## Supporting information


Figure S1.
Figure S2.
Figure S3.
Figure S4.

## Data Availability

The dataset used in this study was obtained for clinical purposes. Data can be shared for research purpose upon reasonable request and after approval by the local ethics committee.
