# Supplementary material for: Asymmetry of generalized discharges in idiopathic generalized epilepsy in adults
Source: Epilepsia. 2025 Jun 21;66(8):e202–10. doi: 10.1111/epi.18509 (PMC12371676; doi:10.1111/epi.18509)
Supplement: Supplementary file 1 — Figure S1. Figure S2. Figure S3. Figure S4. [file EPI-66-e202-s001.docx]

**Asymmetry of generalized discharges in**

**idiopathic generalized epilepsy in adults**

Joao **Pizarro**, Matthew C **Walker**, Laurent **Sheybani**

**Supplementary Information**

**Results**

**Epidemiological data**

Sixty-two patients were included (45 females, median age [range]: 26-year-old [16-63]). We included a mean ± SD of 37 ± 65 GEDs per patient during sleep and 48 ± 123 GEDs during wakefulness. Data were not available, or patients did not display GEDs during sleep and wake in 8 and 5 cases respectively. All patients displayed ≥ 1 GEDs during sleep or wake.

**Right-left asymmetry**

When including only people with definite IGE, we found no effect of condition, or interaction with vigilance (main effect of condition: F(1.3,56.5)=3.2, *p*=0.07; condition * vigilance interaction: F(1.4,44,6)=0.81, *p*=0.41, Supplementary Fig. 3).


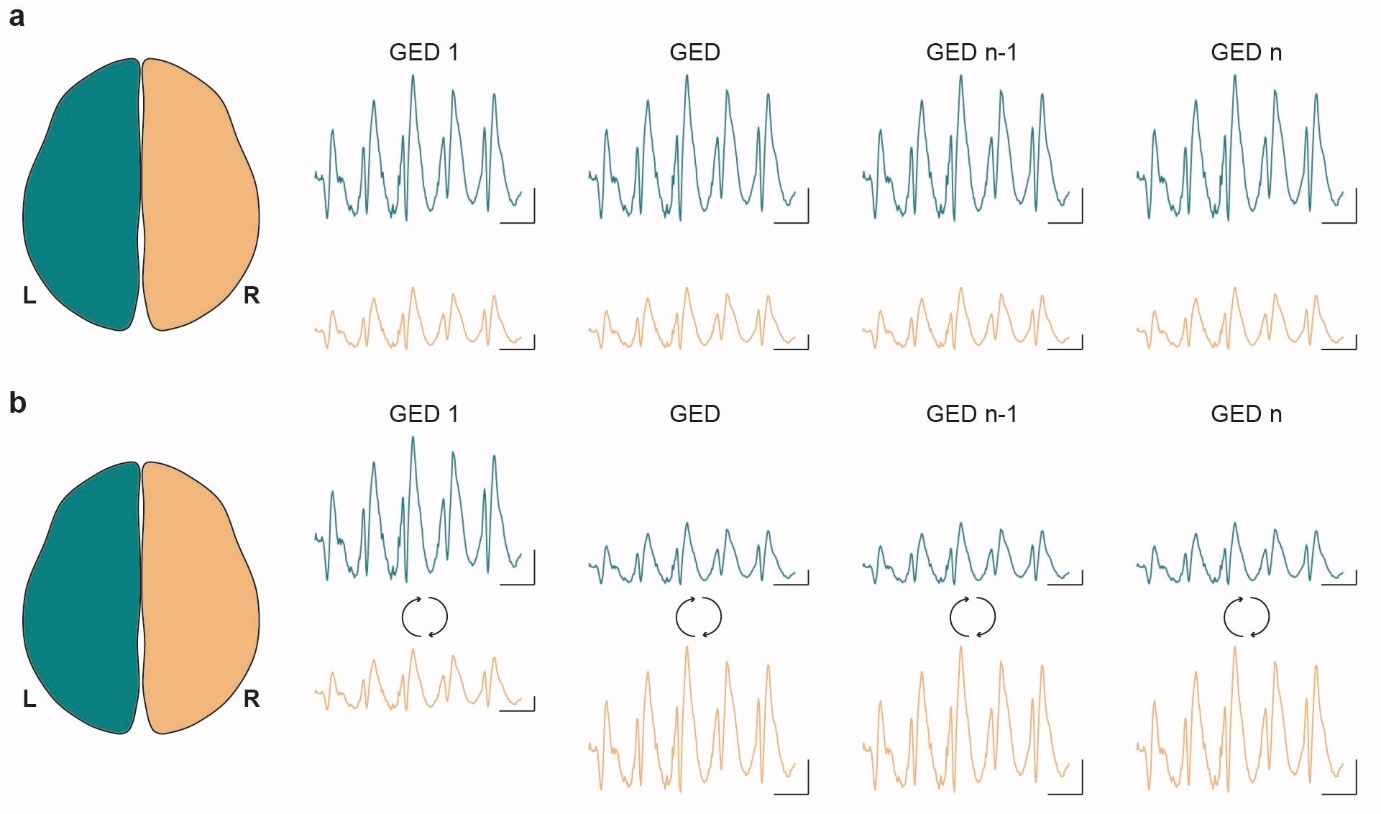


**Supplementary Figure 1 | Computing asymmetry of GEDs**

**(a)** By default, we compute the ratio of GED amplitude in the right divided by the left hemisphere. In this example, the hemispheric asymmetry would thus be < 1. We thus flip the ratio to 1 / ratio. **(b)** Computation of control ratios follows the same strategy to that of true ratios. According to the null hypothesis, whether the ratio is computed as left to right or right to left amplitude does not change the amplitude of the ratio. Across 10,000 permutations, we thus compute left / right and right / left ratios. The circled arrows reflect the fact that assigning the left and right hemisphere to the numerator or denominator of the ratio is done randomly. At each permutation, we flip the ratio to be > 1. In this example permutation, the surrogate right to left ratio is > 1 and thus does not need to be flipped. We then calculate the average control ratio across these permutations and compare it to the actual ratio. A systematic bias for GEDs to be higher in one hemisphere would thus be reflected in higher asymmetry of the actual GEDs ratios. Scale: 50 µV, 200 ms for (a) and (b).


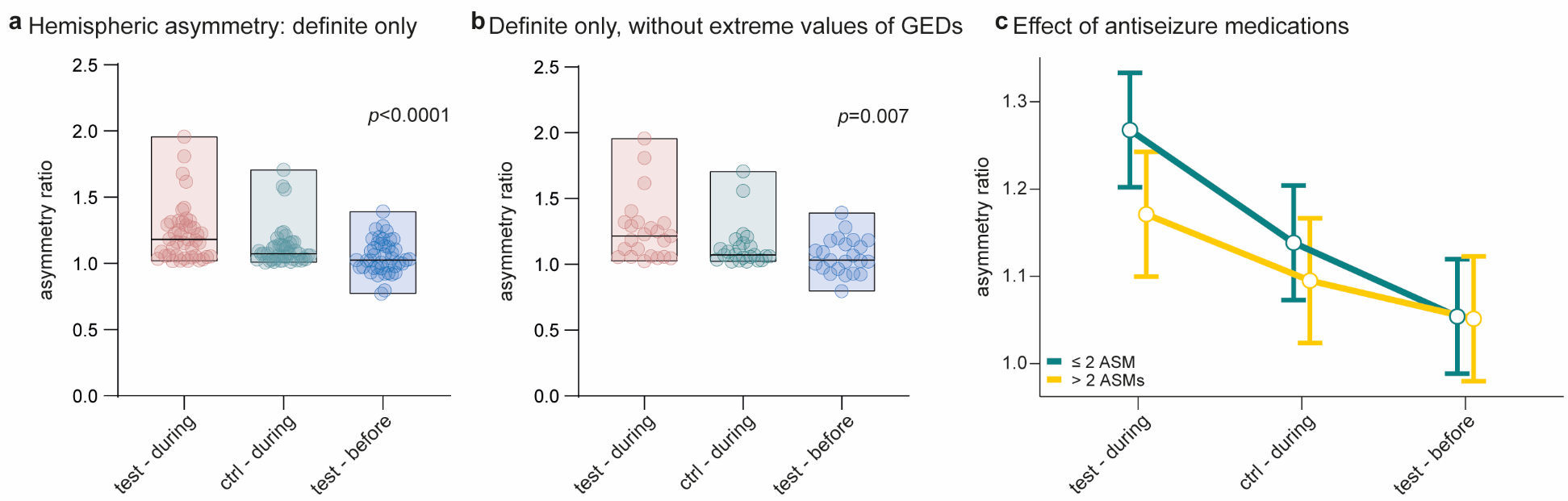


**Supplementary Figure 2 | Asymmetries in definite IGE cases**

We obtained similar results when including only definite cases of IGE for the computation of the **(a)** hemispheric asymmetry and **(b)** hemispheric asymmetry after excluding patients with extreme number of GEDs. *P*-values indicate the main effect of condition. For display, data are combined across sleep and wake given the lack of effect of vigilance. Boxes: min to max, line at median. Dots: individual subjects. **(c)** The number of ASMs presents no association with the degree of hemispheric asymmetry. Whiskers: 95% confidence interval


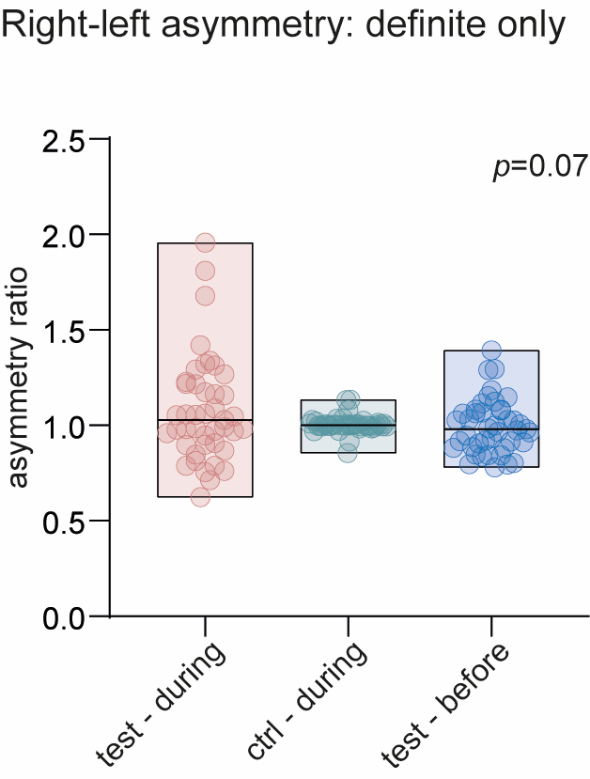


**Supplementary Figure 3 | Right-left asymmetry in definite cases**

Similar to observation on probable and definite cases altogether, we found no right-left asymmetry on definite cases of IGE.

**
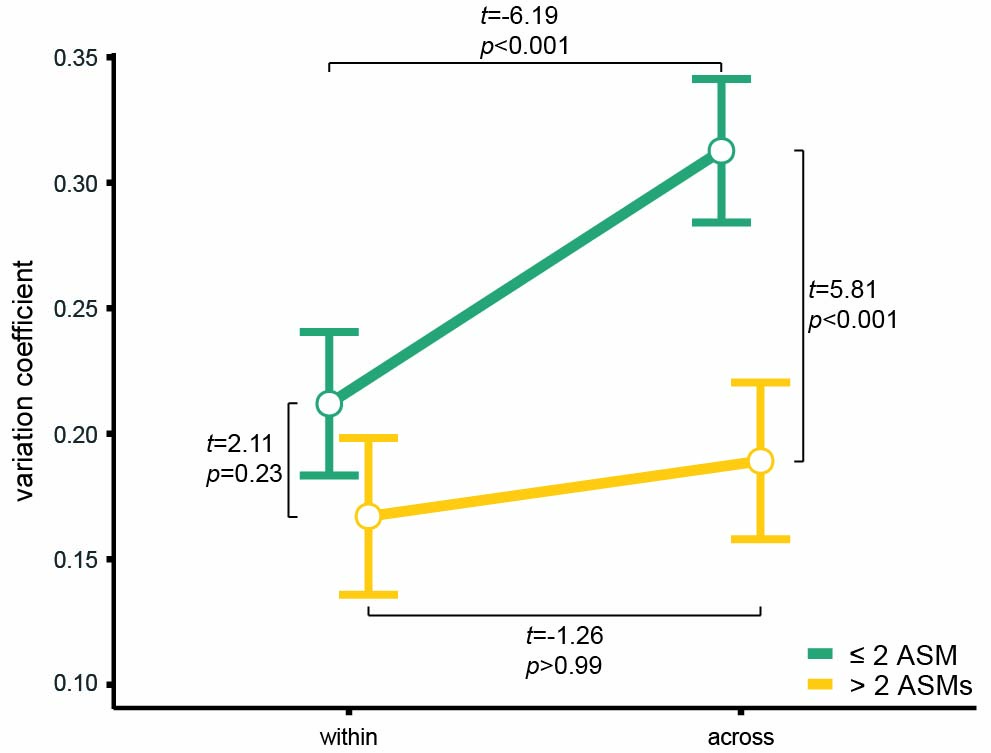
**

**Supplementary Figure 4 | Number of ASMs is associated with different asymmetry consistency**

Asymmetry of GEDs in people with > 2 ASMs is more homogenous than those with ≤ 2 ASMs and the within-patients consistency is thus not significantly lower than that of the across-patients consistency in people with > 2 ASMs (mean difference, standard error: -0.02, ±0.02, *t*=-1.26, *p*>0.99). In contrast, people with ≤ 2 ASMs have more heterogeneous EEG lateralisation and, consequently, the within-patients consistency is significantly lower than that of the across-patients consistency (-0.10, ± 0.02, *t*=-6.19, *p*<0.001). Whiskers: 95% confidence interval.
